# Experimental evaluation and computational modeling of the effects of encapsulation on the time-profile of glucose-stimulated insulin release of pancreatic islets

**DOI:** 10.1186/s12938-015-0021-9

**Published:** 2015-03-28

**Authors:** Peter Buchwald, Sirlene R Cechin, Jessica D Weaver, Cherie L Stabler

**Affiliations:** Diabetes Research Institute, University of Miami, DRI, 1450 NW 10th Ave (R-134), Miami, FL 33136 USA; Department of Molecular and Cellular Pharmacology, University of Miami, Miller School of Medicine, Miami, FL USA; Biomedical Engineering, University of Miami, Miller School of Medicine, Miami, FL USA; DeWitt-Daughtry Department of Surgery, University of Miami, Miller School of Medicine, Miami, FL USA

**Keywords:** Alginate, Diabetes mellitus, Islet encapsulation, FEM model, Glucose-stimulated insulin release, Hill equation, Islet perifusion

## Abstract

**Background:**

In type 1 diabetic patients, who have lost their ability to produce insulin, transplantation of pancreatic islet cells can normalize metabolic control in a manner that is not achievable with exogenous insulin. To be successful, this procedure has to address the problems caused by the immune and autoimmune responses to the graft. Islet encapsulation using various techniques and materials has been and is being extensively explored as a possible approach. Within this framework, it is of considerable interest to characterize the effect encapsulation has on the insulin response of pancreatic islets.

**Methods:**

To improve our ability to quantitatively describe the glucose-stimulated insulin release (GSIR) of pancreatic islets in general and of micro-encapsulated islets in particular, we performed dynamic perifusion experiments with frequent sampling. We used unencapsulated and microencapsulated murine islets in parallel and fitted the results with a complex local concentration-based finite element method (FEM) computational model.

**Results:**

The high-resolution dynamic perifusion experiments allowed good characterization of the first-phase and second-phase insulin secretion, and we observed a slightly delayed and blunted first-phase insulin response for microencapsulated islets when compared to free islets. Insulin secretion profiles of both free and encapsulated islets could be fitted well by a COMSOL Multiphysics model that couples hormone secretion and nutrient consumption kinetics with diffusive and convective transport. This model, which was further validated and calibrated here, can be used for arbitrary geometries and glucose stimulation sequences and is well suited for the quantitative characterization of the insulin response of cultured, perifused, transplanted, or encapsulated islets.

**Conclusions:**

The present high-resolution GSIR experiments allowed for direct characterization of the effect microencapsulation has on the time-profile of insulin secretion. The multiphysics model, further validated here with the help of these experimental results, can be used to increase our understanding of the challenges that have to be faced in the design of bioartificial pancreas-type devices and to advance their further optimization.

**Electronic supplementary material:**

The online version of this article (doi:10.1186/s12938-015-0021-9) contains supplementary material, which is available to authorized users.

## Background

To maintain glucose homeostasis, patients with type 1 diabetes mellitus (T1D) require continuous glucose monitoring and exogenous insulin treatment due to the autoimmune destruction of their insulin-producing β-cells. In healthy humans, these β-cells, which are located in pancreatic islets, provide a finely-tuned glucose-insulin control system that maintains blood glucose levels within a relatively narrow range (typically 4–5 mM and usually within 3.5–7.0 mM, i.e., 60–125 mg/dL in fasting subjects) [1,2]. For T1D patients, exogenous insulin delivered via injections or pumps can provide a solution; however, because these therapies cannot fully mimic the inherently complex function of the endocrine pancreas, they are ultimately inadequate, leading to chronic and degenerative complications [3].

Transplantation of pancreatic islet cells can normalize metabolic control in a manner that is not achievable with exogenous insulin. This approach is being explored as an experimental therapy in a select cohort of patients [4,5]. One of the primary challenges impacting islet graft survival in these patients is the compounded immune and autoimmune response to the graft, necessitating life-long immunosuppression that comes with a host of adverse side effects. The encapsulation of islets using various techniques and materials has been extensively explored as a possible approach to circumvent life-long immunosuppression [6-10]. One of the most commonly used biomaterials for such purposes is alginate. It is an algae-derived or seaweed-derived anionic polysaccharide comprised of unbranched polymers of 1,4-linked β-D-mannuronic and α-L-guluronic acid residues, which form a gel in the presence of multivalent cations such as Ca^2+^ or Ba^2+^. Such approaches have their own challenges: for example, failed clinical and preclinical attempts [11,12] make it clear that minimizing the volume of encapsulating material and the corresponding diffusional limitations are crucial for graft success.

Within this context, quantitative models that assist in predicting and understanding the islet microenvironment needed for proper functioning are of considerable relevance to the optimization of future designs. Furthermore, models that can describe the glucose–insulin regulatory system are of obvious general interest. Several have been developed and some are also used in clinical practice, for example, to estimate glucose effectiveness and insulin sensitivity from intravenous glucose tolerance tests (IVGTT) [13]. While some models of insulin release from encapsulated islets have been published [14-20], none of them allow the coupling of both convective and diffusive transport with reactive rates for arbitrary geometries. Most of these models allowed transport by diffusion only, and just a couple incorporated convective transport (i.e., fluid dynamics to model flow) [15,18], and even these were restricted by having to assume cylindrical symmetry. We have recently developed a finite element method (FEM)–based glucose-insulin model for avascular islets that uses an unrestricted approach to couple local (i.e., cellular level) hormone release and nutrient consumption rates with mass transport by convection and diffusion. It can be used for arbitrary geometries including those with flowing fluid phases to calculate insulin output in response to arbitrary incoming glucose profiles [21]. Furthermore, the model can account for both first-phase and second-phase insulin responses, as well as for the glucose-dependence and oxygen-dependence of insulin release. In avascular islets, hypoxia due to oxygen diffusion limitations is likely a major limiting factor [22-27]. Therefore, it is important to incorporate this aspect into the glucose–insulin response model. As our model is particularly well-suited to describe the behavior of regular or encapsulated islets in glucose-stimulated insulin release (GSIR) perifusion experiments, we performed parallel perifusion experiments with rodent islets and frequent sampling (every minute) to quantify the effect that microencapsulation has on the time-profile of insulin secretion and used the results to further calibrate and validate our FEM-based computational model.

## Methods

### Islet isolation and encapsulation

All animal studies were reviewed and approved by the University of Miami Institutional Animal Care and Use Committee. All procedures were conducted according to the guidelines of the Committee on Care and Use of Laboratory Animals, Institute of Laboratory Animal Resources (National Research Council, Washington DC). Animals were housed in microisolated cages in Virus Antibody Free rooms with free access to autoclaved food and water at the Department of Veterinary Resources of the University of Miami. The Preclinical Cell Processing and Translational Models Core at the Diabetes Research Institute performed the rodent islet isolations. Islets were obtained from donor mice (BALB/C, Jackson Lab, Bar Harbor, ME) via mechanically enhanced enzymatic digestion followed by density gradient purification, as previously described [28,29]. Islet purity was assessed by dithizone (Sigma-Aldrich) staining, and islets were counted and scored using a standard algorithm for the calculation of 150 μm diameter islet equivalent (IEQ) number [30,31]. Islets were cultured in completed CMRL 1066-based medium (Mediatech), which is CMRL 1066 supplemented with 10% fetal bovine serum (FBS; Sigma), 20 × 10^−3^ M Hepes Buffer, 1% penicillin-streptomycin (Sigma-Aldrich), and 1% L-glutamine (Sigma-Aldrich). Islets were cultured for 48 h prior to encapsulation.

For encapsulation, standard Ba-Alg microbeads were fabricated using a modification of the protocol originally developed by Lim and Sun [32]. As previously published, 1.6% (w/v) alginate (UP-MVG, Mw = 300 kDa, Mw/Mn = 1.87, DPn of 28, Batch # FP-504-03, Pronova Novamatrix, FMC) dissolved in phosphate-buffered solution (PBS, Mediatech) was homogeneously mixed with pelleted mouse islets prior to extrusion through a microbead generator (Biorep Technologies, Inc., Miami, FL) into a crosslinking bath of 1.5% (w/v) barium chloride, supplemented with 10 mM 3-(N-morpholino)propanesulfonic acid (MOPS), 140 mM D-Mannitol, and 0.025% Tween-20. After crosslinking (10 min), microbeads were washed with PBS and cultured for 48 h prior to study. Resulting microbeads were an average size of 800 μm.

### Islet perifusion

The perifusion experiments (dynamic glucose-stimulated insulin release, GSIR) were performed using a custom built apparatus that allows parallel perifusion in up to eight channels (Biorep). Fifty IEQ of unencapsulated or encapsulated islets were handpicked and loaded in Perspex microcolumns, between two layers of acrylamide-based microbead slurry (Bio-Gel P-4, Bio-Rad Laboratories, Hercules, CA). Perifusing buffer containing 125 mM NaCl, 5.9 mM KCl, 1.28 mM CaCl_2_, 1.2 mM MgCl_2_, 25 mM HEPES, 0.1% bovine serum albumin, and 3 mM glucose at 37°C with selected glucose (low = 3 mM; high = 11 mM) or KCl (25 mM) concentrations was circulated through the columns at a rate of 100 μL/min. After 45–60 minutes of washing with the low glucose solution for stabilization, islets were stimulated with the following sequence: 5 min of low glucose, 20 min of high glucose, 15 min of low glucose, 5 min of KCl, and 10 min of low glucose. Serial samples (100 μL) were collected every minute from the outflow tubing of the columns in an automatic fraction collector designed for a multi-well plate format. The sample container harboring the islets and the perifusion solutions were kept at 37°C in a built-in temperature controlled chamber. The perifusate in the collecting plate was kept at <4°C to preserve the integrity of the analytes. Insulin concentrations were determined with a commercially available ELISA kit (Mercodia Inc., Winston Salem, NC). To account for possible differences in viability/purity across experiments as well as in IEQ numbers among islets in different channels, values were rescaled by up to 30% taking into account the KCl-induced release as a normalization factor for each condition. Data used here are averages of duplicate samples for both free and encapsulated islets perifused in parallel from three independent experiments.

### Computational methods

**Figure 1:**
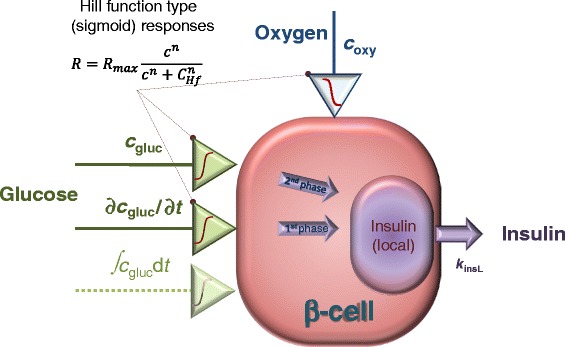
**Basic concept of the present model of insulin release in β-cells.** Response is determined by the local glucose concentration, *c*_gluc_, and its rate of change, ∂*c*_gluc_/∂*t*, but it is also influenced by the local oxygen concentration, *c*_oxy_.

For computational modeling, our previously developed local concentration-based insulin secretion model has been used. A conceptual schematic of the model is shown in Figure 1; a detailed description of its implementation and parameterization can be found in [21] (see also Additional file 1: Appendix 1). Here, only a brief summary is included to permit interpretation and understanding of the results. A total of four concentrations were used for (convective and diffusive) mass transport modeling, with their corresponding equations (application modes): glucose, oxygen, and ‘local’ and released insulin (*c*_gluc_, *c*_oxy_, *c*_insL_, *c*_ins_). Diffusion was assumed to be governed by the generic diffusion equation in its nonconservative formulation (incompressible fluid):

1 $$ \frac{\partial c}{\partial t}+\mathit{\nabla}\cdot \left(-D\nabla c\right)=R-\mathbf{u}\cdot \mathit{\nabla}c $$

where, *c* denotes the concentration [mol⋅m^−3^] and *D* the diffusion coefficient [m^2^⋅s^−1^] of the species of interest, *R* the reaction rate [mol⋅m^−3^⋅s^−1^], **u** the velocity field [m⋅s^−1^], and ∇ the standard *del* (*nabla*) operator ($\mathit{\nabla}\equiv \mathbf{i}\frac{\partial }{\partial x}+\mathbf{j}\frac{\partial }{\partial y}+\mathbf{k}\frac{\partial }{\partial z}$). Diffusion coefficients (*D*) used were the same as in the original model [21]; they were selected as consensus estimates of values available from the literature. Values in the present implementation for water, tissue (islet), and alginate (capsule) are as follows (all in m^2^⋅s^−1^) oxygen: 3.0 × 10^−9^, 2.0 × 10^−9^, 2.5 × 10^−9^; glucose: 0.9 × 10^−9^, 0.3 × 10^−9^, 0.6 × 10^−9^; and insulin: 0.15 × 10^−9^, 0.05 × 10^−9^, 0.1 × 10^−9^.

As an important part of the model, all consumption and release rates were assumed to follow Hill–type dependence (generalized Michaelis-Menten kinetics) on the local concentrations as this provides a convenient and easily parameterizable mathematical function:

2 $$ R={f}_H(c)={R}_{\max}\frac{c^n}{c^n+{C}_{Hf}^n} $$

Parameters here were *R*_max_, the maximum reaction rate [mol⋅m^−3^⋅s^−1^], *C*_Hf_, the concentration corresponding to half-maximal response [mol⋅m^−3^], and *n*, the Hill slope characterizing the shape of the response. The parameter values used for the different release and consumption functions (i.e., insulin, glucose, oxygen; e.g., *C*_Hf,gluc_, *C*_Hf,oxy_, etc.) were different [21]; their values used in the model are summarized in Table 1.

**Table 1:** **Summary of Hill function parameters used in the present model (Figure** 1 **; detailed equations in** [21]**)**

**Rate**	**Var.**	***C***_Hf_	***n***	***R***_max_	**Property and comments**
*R*_oxy_	*c*_oxy_	1 μM	1	−0.034 mol/m^3^/s	Oxygen consumption, base. Cut to 0 below critical value, *c*_oxy_ < *C*_cr,oxy_.
*R*_oxy_	*c*_gluc_	7 mM	2.5	N/A	Oxygen consumption, *φ*_o,g_ metabolic part. Due to increasing metabolic demand. Parallels second-phase insulin secretion rate.
*R*_gluc_	*c*_gluc_	10 μM	1	−0.028 mol/m^3^/s	Glucose consumption. Contrary to oxygen, has no significant influence on model results.
*R*_ins,ph2_	*c*_gluc_	7 mM	2.5	3 × 10^−5^ mol/m^3^/s	Insulin secretion rate, second-phase. Total secretion rate is modulated by local oxygen availability (last row).
*R*_ins,ph1_	∂*c*_gluc_/∂*t*	0.03 mM/s	2	21 × 10^−5^ mol/m^3^/s	Insulin secretion rate, first-phase. Modulated to have maximum sensibility around *c*_gluc_ = 5 mM and be limited at very large or low *c*_gluc_.
*R*_ins_, *φ*_o,g_	*c*_oxy_	3 μM	3	N/A	Insulin secretion rate, *φ*_o,g_ oxygen dependence. Limits insulin secretion if *c*_oxy_ becomes critically low.

For avascular islets, oxygen availability is a main limiting factor because the solubility of oxygen in culture media or in tissue is much lower than that of glucose; hence, available oxygen concentrations are usually much more limited [21,26]. To account for the increased metabolic demand of insulin release and production at higher glucose concentrations, the model also included a dependence of the oxygen consumption (*R*_oxy_) on the local glucose concentration via a modulating function *φ*_*o,g*_(*c*_gluc_) (see [21] for further details). The core of the model was the functional form describing the glucose-dependence of the insulin secretion rate, *R*_ins_. For this purpose, a combination of two sigmoid Hill functions was used. One describes the glucose-insulin dynamics of the second-phase response as a function of the local glucose concentration, *c*_gluc_, with the help of parameters shown in the *R*_ins,ph2_ row of Table 1. The other describes the first-phase response as a function of the (local) change in glucose concentration, i.e., glucose time-gradient (*c*_*t*_ = ∂*c*_gluc_/∂*t*) with the help of parameters shown in the *R*_ins,ph1_ row of Table 1. This was non-zero only when the glucose concentration increased, i.e., only when *c*_t_ > 0. We found that for a correct time-scale of insulin release, an additional ‘local’ insulin compartment had to be added (Figure 1) so that insulin was assumed to be first secreted into this compartment and then released from here following a first order kinetics, d*c*_insL_/d*t* = *R*_ins_ − *k*_insL_(c_insL_ − *c*_ins_); for details, see [21]. The original model, which was calibrated for human islets, used a corresponding rate constant of *k*_insL_ = 0.003 s^−1^. Here, to fit the data obtained with murine islets, this was increased to 0.006 s^−1^ to have a slightly faster release; this was the only parameter modified compared to the original model.

Finally, to incorporate media flow, these convection and diffusion models were coupled to a fluid dynamics model. For this purpose, the incompressible Navier–Stokes model for Newtonian flow (constant viscosity) was used to calculate the velocity field **u** resulting from convection [33]:

3 $$ \begin{array}{c}\hfill \rho \frac{\partial \mathbf{u}}{\partial t}-\eta {\mathit{\nabla}}^{{}^2}\mathbf{u}+\rho \left(\mathbf{u}\cdot \mathit{\nabla}\right)\mathbf{u}+\mathit{\nabla}p=\mathbf{F}\hfill \\ {}\hfill \mathit{\nabla}\cdot \mathbf{u}=0\hfill \end{array} $$

The first equation is the momentum balance; the second is the equation of continuity for incompressible fluids. Standard notation was used with *ρ* denoting density [kg⋅m^−3^], *η* viscosity [kg⋅m^−1^⋅s^−1^ = Pa⋅s], *p* pressure [Pa, N⋅m^−2^, kg⋅m^−1^ ⋅s^−2^], and **F** volume force [N⋅m^−3^, kg⋅m^−2^⋅s^−2^]. The flowing media was assumed to be an essentially aqueous media at physiological temperature (37°C). Incoming media was assumed to be in equilibrium with atmospheric oxygen and, thus, to have an oxygen concentration of *c*_oxy,in_ = 0.200 mol⋅m^−3^ (mM) corresponding to *p*_O2_ ≈ 140 mmHg. For physiologically relevant conditions, lower values have to be used as tissue oxygen concentrations are likely to be around only *c*_oxy,tis_ = 0.060 mol⋅m^−3^ (mM) corresponding to *p*_O2_ ≈ 40 mmHg.

The model was implemented in COMSOL Multiphysics 4.4 (COMSOL Inc., Burlington, MA) and solved as a time-dependent (transient) problem, allowing intermediate time-steps for the solver, as described previously. Mesh and boundary conditions used were also as described before [21] (see also Additional file 1: Appendix 1, Additional files for further details). Similar to our previous models of human islet perifusions [21,34], here, we also used two spherical islets of 100 and 150 μm diameter placed in a 2D cross-section of a cylindrical tube with fluid flowing from left to right. Islets were considered homogeneous inside; individual cells (e.g., α- or β-cells) were not considered separately. Islet sizes were selected based on our analysis of the size distribution of isolated islets [31]. This confirmed that the expected value of islet diameter is 95 μm, whereas the expected value of islet volume is 1.2 × 10^6^ μm^3^, corresponding to the volume of an islet with *d* = 133 μm [31].

## Results and discussion

**Figure 2:**
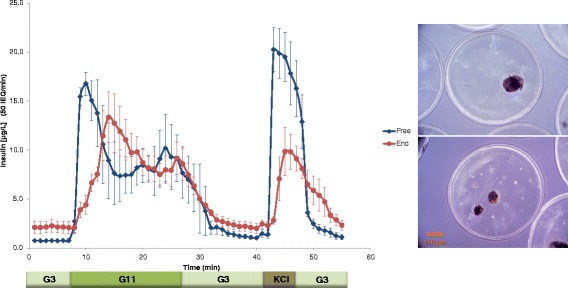
**Glucose-induced insulin secretion in unencapsulated (free) and encapsulated islets (blue diamonds and red dots, respectively).** Average of experimental data for free and alginate-encapsulated mouse islets perifused in parallel using a low (3 mM; G3) → high (11 mM; G11) → low (G3) incoming glucose stimulation (plus 5 min KCl followed by G3), as shown below the *x*-axis. Data represent the average ± SE for three experiments performed in duplicate with ~ 50 IEQ per channel. Representative encapsulated islets used in these experiments are shown on the right.

To accurately quantify the effect that microencapsulation has on the time-profile of insulin secretion from isolated islets, we first performed high-resolution parallel perifusion experiments with unencapsulated and alginate-encapsulated murine islets. Then, we used the results to validate and further calibrate our FEM-based glucose-insulin computational model. Perifusion was performed with four channels in parallel and using a low (3 mM), high (11 mM), low (3 mM) glucose step with frequent sample collection (every minute); the insulin response obtained is shown in Figure 2. To allow a clear delineation of the first-phase response, the high glucose step (G11) was maintained for 20 min, which is longer than in our previously used standard protocols [34–36]. Unencapsulated islets demonstrated a well-defined first-phase peak, followed by a second-phase plateau, with possibly a slightly rising tendency (Figure 2). This was expected, given normal functioning islets release insulin in a biphasic manner in response to a stepwise increase of glucose (e.g., a relatively quick transient spike of 5–10 min /first phase/ followed by a sustained second phase that is slower and somewhat delayed [37–40]). Responses from the encapsulated islets were similar, but slightly delayed and blunted. This was again expected, due to the impact of the encapsulating hydrogel and agrees with published reports [32,41,42].

**Figure 3:**
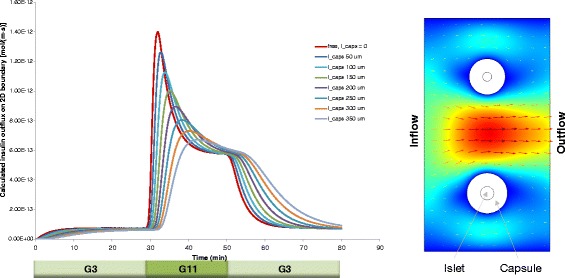
**Model-predicted effect of capsule thickness on GSIR perifusion.** Calculated insulin outflow is shown in response to a stepwise glucose stimulation with the current computational model for two encapsulated islets (*d* = 100 & 150 μm) as a function of increasing capsule thickness (*l*_caps_) from 0 (free islets) and 50 to 350 μm. Shown on the right is an illustration of the main geometric setup used for the current model with fluid flow from left to right, and 100 (top) and 150 (bottom) μm islets.

**Figure 4:**
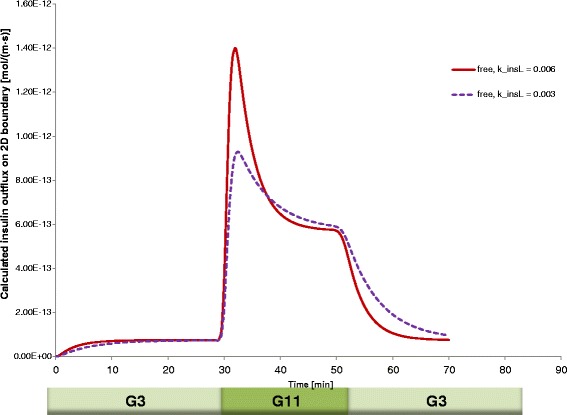
**Effect on the insulin release profile of the single parameter adjustment used (** ***k***_insL_ **= 0.006 s** ^**−1**^ **vs. the original 0.003 s** ^**−1**^ **) to fit release of murine islets with the model previously parameterized for human islets (dark red vs. dashed purple lines, respectively).**

**Figure 5:**
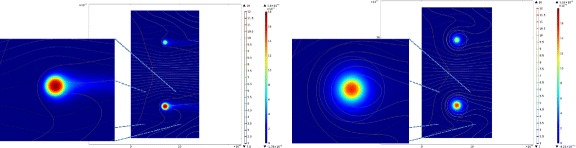
**Model-calculated insulin concentrations in response to increasing glucose concentrations.** Calculated insulin concentrations for two illustrative free and encapsulated islets (left and right, respectively; *d* = 100 (top) & 150 μm (bottom), *l*_caps_ = 150 μm) under normoxic conditions. Data shown as surface plot are insulin concentration (color-coded from blue for low to red for high. Streamlines show the flow of the perifusion fluid (color-coded for velocity; flow from left to right) and colored contour lines show isolevels for the perifusing glucose (from light blue for low to light red for high). Model calculated values are shown during the increase of the glucose concentration from 3 mM to 11 mM; first phase response is noticeably delayed and blunted in the encapsulated islet.

Another important goal of the present work was to verify if these data can be fitted by our recently developed complex FEM-based computational model [21]. This local concentration-based model predicted a similar behavior for hydrogel-encapsulated islets. Figure 3 shows the theoretical predicted values for a setup mimicking the present experimental conditions. Two perifused islets that are in the center of spherical capsules were used in the model, and the capsule thickness was varied from 50 to 350 μm. The model assumes that the insulin-secreting β-cells act as sensors of both the local glucose concentration and its change. Hence, a first-phase response related to the change in (local) glucose concentration as well as a second-phase response related to the (local) glucose concentration is incorporated [21]. Insulin is released within the islets following Hill–type sigmoid response-functions of the local (i.e., cellular level) glucose concentration, *c*_gluc_, and its time-gradient, ∂*c*_gluc_/∂*t*, resulting in second– and first–phase insulin responses, respectively (Figure 1). Oxygen and glucose consumption by the islet cells are incorporated in the model using Michaelis-Menten–type kinetics (i.e., Hill equation with *n* = 1). Since lack of oxygen (hypoxia) can be an important limiting factor in avascular islets [26], oxygen concentrations are also allowed to limit the rate of insulin secretion, following again a Hill–type equation. Finally, all the local (cellular-level) oxygen, glucose, and insulin concentrations are combined together with solute transfer equations to calculate observable, external concentrations as a function of time and incoming glucose and oxygen concentrations (see Computational methods and Additional file 1: Appendix 1 for further details). Calculations were done using the same model parameterized originally based on perifusion data from human islets [21], except the kinetics of insulin release was increased slightly (*k*_insL_ = 0.006 s^−1^ vs. the original 0.003 s^−1^) to account for the somewhat sharper first phase response of murine islets observed here as compared to human islets [34,43]. The effect of this change on the predicted insulin release profile of free islets is shown in Figure 4. An important advantage of this model is that it allows for calculation of the distribution of all concentrations of interest at any time-point during the perifusion; an illustrative example of insulin concentration during the increase of incoming glucose concentration is shown in Figure 5 comparing the response of free and encapsulated islets.

**Figure 6:**
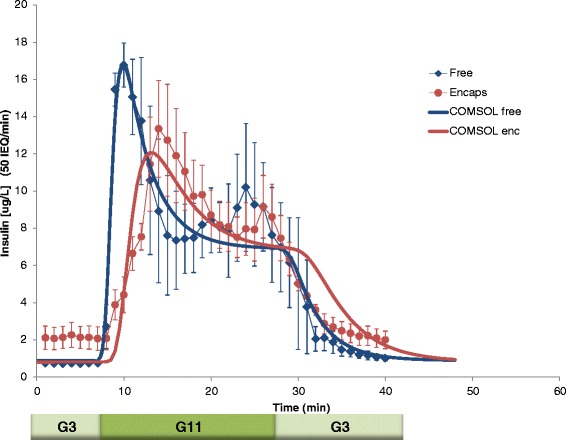
**Fit of the experimental glucose-induced insulin secretion in unencapsulated (free) and encapsulated islets perifused in parallel with the computational model.** Experimental data are the same as in Figure 2; calculated insulin outflow in response to the same stepwise glucose is with the current model for capsule thicknesses of 0 (free islets) and *l*_caps_ = 150 μm assuming islet sizes as shown in Figure 5 and following a 2D to 3D conversion with a total islet volume scaled to 50 IEQ (see text).

Obtained experimental insulin-release profiles could be fitted well with the present model both for the free and encapsulated islets (*r*^2^ of 0.952 and 0.853, respectively) (Figure 6). Remarkably, the total calculated outgoing flux, which requires scaling with height data to convert the 2D flux to 3D values, gave the present fit shown in Figure 6 after scaling to 50 IEQ (i.e., an islet volume of 50 × 1.77·10^−13^ m^3^) – exactly the islet equivalent (IEQ) number used in the experiments. This was particularly encouraging as it supports the accuracy of the insulin secretion rate parameter values, *R*_ins_ (maximum reaction rate) and *C*_Hf,ins_ (the concentration corresponding to half-maximal response) (Table 1), originally derived from human data, and indicates that they can give an adequate description for murine islets as well (with the single adjustment of increasing the *k*_insL_ value, see Figure 4).

Predicted responses for encapsulated islets depend on the thickness of the spherically symmetric capsules assumed for the calculation (Figure 3). The fit shown in Figure 6 was obtained with a capsule size of *l*_caps_ = 150 μm, which gave the lowest sum of squared errors (SSE). Minimum SSE for the fitting was obtained with a capsule size of *l*_caps_ = 150 μm and an IEQ of 50.6. While this thickness might be smaller than the average half-distance for centrally located islets within these 800 μm diameter microbeads (see inset in Figure 2), it is not unrealistic considering the asymmetric position of most islets within the capsule and that the most efficient exchange will take place along the shortest diffusional path to the bead surface. Lacking corresponding experimental data, the effects of different alginate concentrations were not modeled. However, they are expected to modify the diffusivities within the capsule and, ultimately, result in effects that are to a good extent similar to those caused by changes in capsule thickness.

To allow acceptable computation times with adequate time-sampling along the entire perifusion interval (~60 min), the present computational model is implemented as a 2D model (Figure 3). However, we have shown previously that 2D models represent well the results of the much more time consuming 3D models [21,26]. Another advantage of the general nature of the present FEM model is that it allows easy expansion of the model from simple passive diffusion within the capsule to incorporation of convective flow of the aqueous media through the hydrogel capsule, which has been considered a possibility in a few cases (e.g., [44,45]). This can be implemented by permitting flow, but with increased viscosity, within the capsule. We did a brief exploration with such an extended model on the impact of different viscosities for the flow within the encapsulating material on the overall pattern of flow and the predicted insulin profile (data not shown), but we found them too similar to those of the diffusion-only model to allow distinguishing between the models without further knowledge on the parameters (i.e., viscosities vs. diffusion coefficients).

**Figure 7:**
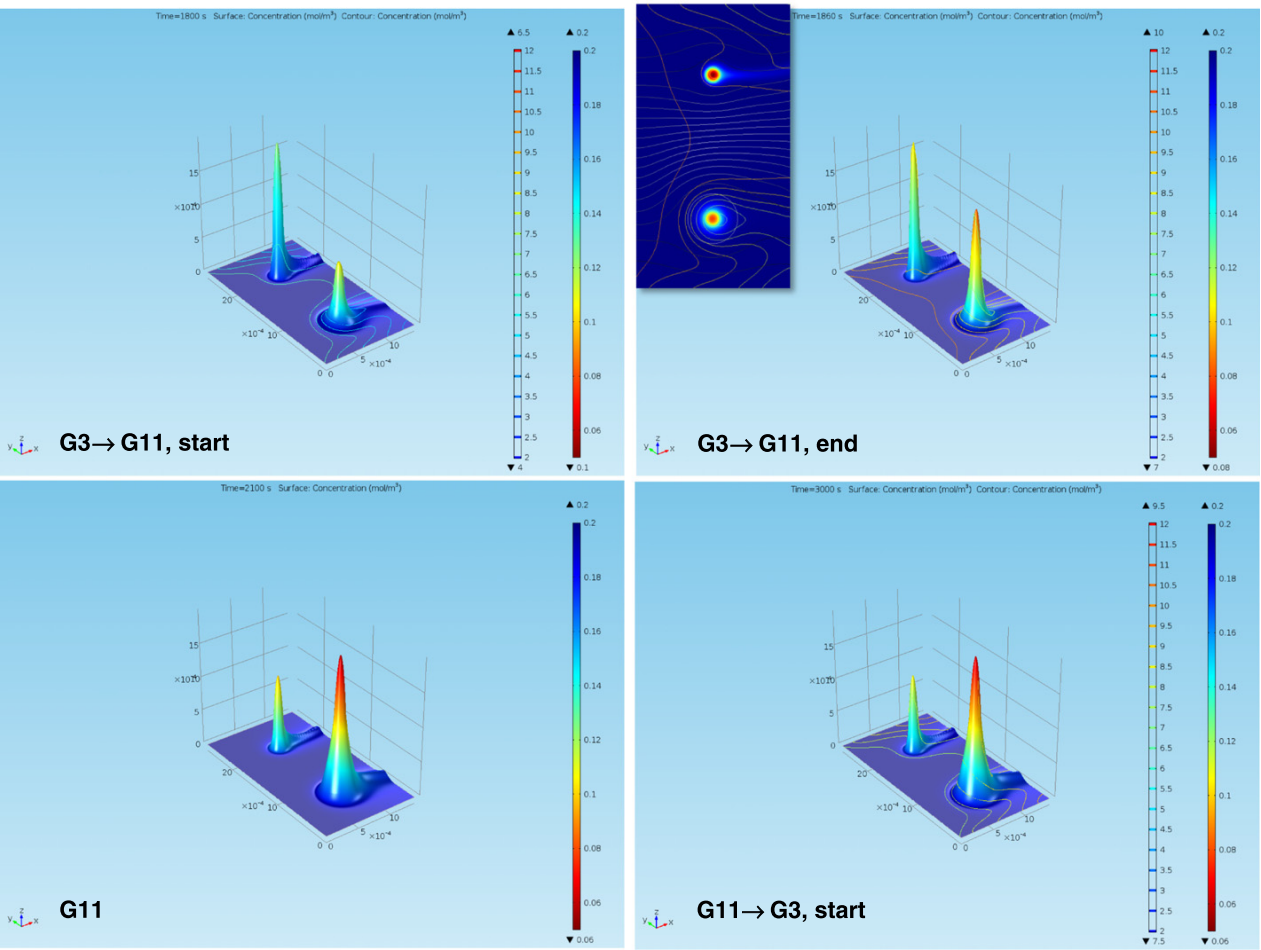
**Model-predicted delay in insulin response and increased hypoxia for encapsulated islets shown as three-dimensional surfaces.** Side-by-side comparison of the GSIR response of free and encapsulated islets (both having *d* = 150 μm; encapsulated islet on the right side) using a 3D surface representation of insulin (height data) and oxygen (color code; blue = high; red = low). The corresponding 2D figure is included for reference (inset).

To better highlight the difference in their dynamic response, we also performed modeling of a free and an encapsulated islet side-by-side within the same chamber. Responses at illustrative time-points (e.g., during the increase in glucose concentration) are shown in Figure 7, with corresponding animations included as Additional files 2 and 3. Of note, the present experiments were performed at atmospheric oxygen (*p*_O2_ = 140 mmHg; *c*_oxy_ = 0.20 mM), while some other published perifusion experiments were performed using oxygen-enriched perifusion media (e.g., 95% O_2_) to minimize the effects of oxygen limitation. At lower oxygen concentrations, such as those mimicking the tissue oxygen concentrations that transplanted islets are likely to encounter (e.g., *p*_O2_ = 35–45 mmHg; *c*_oxy_ = 0.05–0.065 mM), the loss in insulin secreting ability is likely to be more significant as the encapsulated islets will be impacted more by hypoxia [21]. For example, our calculations with this model under low oxygen conditions predicted that free islets can still secrete insulin at around 70–75% of their normal rate, whereas encapsulated islets can only operate at around 50% of the full rate, and their response is especially hampered at larger glucose levels. To overcome this nutrient diffusion problem, many different approaches have been and are being explored including, for example, use of smaller or more oxygen-permeable capsules, co-encapsulation of anti-inflammatory drugs, use of smaller or more hypoxia-resistant islets, local oxygen delivery near the islets, and others, as reviewed in detail elsewhere [10,12].

## Conclusions

Overall, the high-resolution experimental results collected herein are in agreement with previous results [32,41,42,46], whereby the GSIR response of alginate microencapsulated islets in perifusion experiments was found to be similar to that of free islets, but somewhat delayed and blunted – a difference that increases with increasing capsule thickness. Optimization of encapsulation technologies to reduce the effects of diffusion limitations (i.e., thinner capsules, smaller islets, increased diffusivities, incorporation of oxygen delivery, or improved access to nearby blood-flow) could minimize the delay and blunting of the first-phase response, which could be important as an accelerated loss of the first-phase insulin response has been found in those progressing toward T1D [47]. We found that these GSIR perifusion experiments could be very well modeled by our local concentration-based FEM computational model, further confirming its current parameterization. Because the model uses an unrestricted approach to couple hormone secretion and nutrient consumption kinetics with diffusive and even convective transport, it can be used for arbitrary geometries and glucose stimulation sequences. Thus, it is well suited for the quantitative characterization of the insulin response of cultured, perifused, transplanted, and encapsulated islets under various conditions.

## Additional files

Additional file 1:
**Appendix 1.** Local glucose and oxygen concentration-based finite element method (FEM) used to model the insulin secretion of pancreatic islets (β-cells): Computational method details. Additional file 2:
**Video A1: GlucInsDyn_wHalfEncaps_wParamGeo_Model20_v44_InsulinSurfaceHeight.avi.**
Additional file 3:
**Animated GIF A2: GlucInsDyn_wHalfEncaps_wParamGeo_Model20_v44_InsConc01.gif.** Movie (.avi) and corresponding animated image (.gif) files showing the parallel comparison of the time-course of the GSIR response of free and encapsulated islets (both having *d* = 150 μm; encapsulated islet on the right side) to a glucose step (3 mM → 11 mM → 3 mM). The same 3D surface representation with insulin concentrations as height data and oxygen concentration as color code (blue = high, red = low) is used as in Figure 7.

## Abbreviations

FEM: Finite element method
GSIR: Glucose-stimulated insulin release
IEQ: Islet equivalent
SSE: Sum of squared errors
T1D: Type 1 diabetes mellitus
