# Supplementary material for: Experimental evaluation and computational modeling of the effects of encapsulation on the time-profile of glucose-stimulated insulin release of pancreatic islets
Source: Biomed Eng Online. 2015 Mar 28;14:28. doi: 10.1186/s12938-015-0021-9 (PMC4403786; doi:10.1186/s12938-015-0021-9)
Supplement: Additional file 1: — Appendix 1. Local glucose and oxygen concentration-based finite element method (FEM) used to model the insulin secretion of pancreatic islets (β-cells): Computational method details. [file 12938_2015_21_MOESM1_ESM.pdf]

Peter Buchwald, Sirlene R. Cechin, Jessica D. Weaver, and Cherie L. Stabler:
Experimental Evaluation and Computational Modeling of the Effects of Encapsulation on the Time-Profile of Glucose-Stimulated Insulin Release of Pancreatic Islets

Appendix 1

Local glucose and oxygen concentration-based finite element method (FEM) used to model the insulin secretion of pancreatic islets (β-cells): Computational method details.

All calculations for the present work have been done with our previously developed local concentration-based insulin secretion model [1]. A conceptual summary of this FEM-based model is included in Figure 1, and the computational implementation (COMSOL Multiphysics) is briefly summarized in the Method section; a fully detailed description is presented here.

Mass transport (convective and diffusive)

The model uses a total of four concentrations (species), each with its own corresponding equation (application mode): glucose, oxygen, 'local' insulin, and released insulin (c_{gluc}, c_{oxy}, c_{insL}, and c_{ins}). For each of these species, diffusion is assumed to be governed by the generic diffusion equation in its nonconservative formulation (incompressible fluid) [2, 3]:

$$\frac{\partial c}{\partial t} + \nabla \cdot \left(-D\nabla c\right) = R - \mathbf{u} \cdot \nabla c \qquad \text{(A 1)}$$

where, c denotes the concentration [mol·m^{-3}] and D the diffusion coefficient [m^2·s^{-1}] of the species of interest, R denotes the reaction rate [mol·m^{-3}·s^{-1}], $\mathbf{u}$ the velocity field [m·s^{-1}], and ∇ the standard *del* (*nabla*) operator, $\nabla \equiv \mathbf{i}\frac{\partial}{\partial x} + \mathbf{j}\frac{\partial}{\partial y} + \mathbf{k}\frac{\partial}{\partial z}$ [4]. Diffusion coefficients (D) used are the same as in the original model [1]; they were selected as consensus estimates of values available from the literature. Values in the present implementation are as follows (all in m^2·s^{-1})

Diffusion coefficients, D [m^2·s^{-1}]			
Species \ Media	Water	Tissue (islet)	Alginate (capsule)
Oxygen, D_{oxy}	3.0×10^{-9}	2.0×10^{-9}	2.5×10^{-9}
Glucose, D_{gluc}	0.9×10^{-9}	0.3×10^{-9}	0.6×10^{-9}
Insulin, D_{ins}	0.15×10^{-9}	0.05×10^{-9}	0.1×10^{-9}

Reaction rates

For all species, reaction rates (i.e., consumption and release rates, R) are assumed to follow Hill–type dependence on the local concentrations (i.e., generalized Michaelis-Menten kinetics):

$$R = f_H(c) = R_{\max} \frac{c^n}{c^n + C_{Hf}^n} \qquad (A\ 2)$$

This functional dependence uses three parameters: $R_{\max}$, the maximum reaction rate [mol·m^{-3}·s^{-1}], C_{Hf}, the concentration corresponding to half-maximal response [mol·m^{-3}], and n, the Hill slope characterizing the shape of the response. This function (originally introduced by A. V. Hill [5, 6]) is widely used for biological / pharmacological applications [7] as it provides transition from zero to a limited maximum rate via a smooth an continuously derivable function of adjustable width. The well-known two-parameter Michaelis-Menten equation [8] represents a special case (n = 1) of the Hill equation. Parameter values used for the different release and consumption functions (i.e., insulin, glucose, oxygen; e.g., $C_{\mathrm{Hf,gluc}}$, $C_{\mathrm{Hf,oxy}}$, etc.) are different [1]; values used in the present model are summarized in Table 1. The model has been parameterized [1] by fitting experimental insulin release data from two detailed concentration-dependence perifusion studies: one concentrating on the effect of glucose using isolated human islets [9] and one concentrating on the effect of hypoxia using isolated rat islets [10].

Oxygen consumption

For oxygen consumption, the basic parameter values are maintained from our previous models [1, 11]: $n_{\mathrm{oxy}} = 1$, $R_{\mathrm{max,oxy}} = -0.034$ mol·m^{-3}·s^{-1}, and $C_{\mathrm{Hf,oxy}} = 1$ μM (corresponding to a partial oxygen pressure of $p_{\mathrm{Hf,oxy}} = 0.7$ mmHg) (Table 1). By all indications, the assumption of a regular Michaelis-Menten kinetics (i.e., $n_{\mathrm{oxy}} = 1$) gives an adequate fit. Accordingly, at low oxygen concentrations, where cells only try to survive, oxygen consumption scales with the available concentration c_{oxy} and, at sufficiently high concentration, it plateaus at a maximum ($R_{\max}$). To account for the increased metabolic demand of insulin release and production at higher glucose concentrations, a dependence of R_{oxy} on the local glucose concentration is assumed via a modulating function $\varphi_{o,g}(c_{\mathrm{gluc}})$:

$$R_{oxy} = R_{\max,oxy} \frac{c_{oxy}}{c_{oxy} + C_{Hf,oxy}} \cdot \varphi_{o,g}(c_{gluc}) \cdot \delta(c_{oxy} > C_{cr,oxy}) \qquad (A\ 3)$$

This is done to accommodate experimental indications showing increased oxygen consumption rate in islets when going from low to high glucose (see [1] and references therein). It is assumed that the oxygen consumption rate contains a base-rate and an additional component that increases due to the increasing metabolic demand in parallel with the insulin secretion rate (see eq. A6 later) as a function of the glucose concentration:

$$\varphi_{o,g}\left(c_{gluc}\right)=\phi_{sc}\left(\varphi_{base}+\varphi_{metab}\frac{c_{gluc}^{n_{ins2,gluc}}}{c_{gluc}^{n_{ins2,gluc}}+C_{Hf,ins2,gluc}^{n_{ins2,gluc}}}\right) \tag{A 4}$$

In the present model, the base and metabolic components are assumed to be equal ($\varphi_{\text{base}} = \varphi_{\text{metab}} = 0.5$) and a scaling factor of $\phi_{\text{sc}} = 1.8$ is used [1]. The metabolic component fully parallels that used for insulin secretion ($n_{\text{ins2,gluc}} = 2.5$, $C_{\text{Hf,ins2,gluc}} = 7$ mM; see eq. A6 later). With this selection, oxygen consumption increases about 70% when going from low glucose (3 mM) to high glucose (15 mM). A step–down function, δ, is also used to account for necrosis and cut the oxygen consumption in tissues where the oxygen concentration c_{oxy} falls below a critical value, $C_{\text{cr,oxy}} = 0.1$ μM (corresponding to $p_{\text{cr,oxy}} = 0.07$ mmHg). To ensure a smooth transition, COMSOL Multiphysics' smoothed Heaviside function `flc1hs`, which has a continuous first derivative and no overshoot [12], is used as step-down function, $\delta(c_{\text{oxy}} > C_{\text{cr,oxy}})$ = `flc1hs`($c_{\text{oxy}} - 1.0\times10^{-4}, 0.5\times10^{-4}$).

Glucose consumption

Similar to oxygen consumption, glucose consumption is also assumed to follow simple Michaelis-Menten kinetics ($n_{\text{gluc}} = 1$) with $R_{\text{max,gluc}} = -0.028$ mol·m^{-3}·s^{-1} and $C_{\text{Hf,gluc}} = 10$ μM (Table 1) [1]:

$$R_{gluc}=R_{\max,gluc}\frac{c_{gluc}}{c_{gluc}+C_{Hf,gluc}} \tag{A 5}$$

While these values are draft estimates only, they have only relatively little effect on the calculated insulin secretion. Changes in glucose concentrations due to glucose consumption by islets have only minimal influence on insulin release or cell survival because oxygen diffusion limitations in tissue or in media are far more severe than for glucose [13, 14]. Even if oxygen is consumed at approximately the same rate as glucose on a molar basis and has a three- to four-fold higher diffusion coefficient (see D_{oxy} vs. D_{gluc} values above), this is more than offset by the

differences in the concentrations available under physiological conditions. The solubility of oxygen in culture media or in tissue is much lower than that of glucose making the available oxygen concentrations much more limited (e.g., around 0.05–0.2 mM vs. 3–15 mM assuming physiologically relevant conditions) [14]. Consequently, while oxygen limitations can seriously affect islet function, glucose consumption by islets has only limited effects on the glucose levels reaching the glucose-sensing β-cells.

Insulin release

A crucial part of the model is the function describing the glucose-dependence of the insulin secretion rate, R_{ins}. Glucose (or oxygen) is not a substrate *per se* for insulin production; hence, there is no direct justification for the use of Michaelis-Menten–type enzyme kinetics. Nevertheless, the corresponding generalized form of equation A2 (Hill equation) was found to fit well the experimental results, but a Hill coefficient larger than unity ($n > 1$) is needed because glucose-insulin response is clearly more abrupt than the rectangular hyperbola of the Michaelis-Menten equation corresponding to $n = 1$ [1]. Accordingly, the main function used here to describe the glucose-insulin dynamics of the second-phase response is:

$$R_{ins,ph2} = R_{\max,ins2}\frac{c_{gluc}^{n_{ins2,gluc}}}{c_{gluc}^{n_{ins2,gluc}} + C_{Hf,ins2,gluc}^{n_{ins2,gluc}}} \qquad (A\ 6)$$

with $n_{\mathrm{ins2,gluc}} = 2.5$, $C_{\mathrm{Hf,ins2,gluc}} = 7$ mM, and $R_{\mathrm{max,ins2}} = 3.0\times10^{-5}$ mol·m^{-3}·s^{-1} (Table 1). These values were obtained [1] by fitting the insulin release data of human islets measured by Henquin and co-workers in staircase experiments [9]. $R_{\mathrm{max,ins2}}$ as used here corresponds to a maximum (second phase) secretion rate of ~20 pg/IEQ/min for human islets [9, 15, 16].

The first-phase response is incorporated into the model via a component that depends on the change (time-gradient) of glucose concentration ($c_t = \partial c_{\mathrm{gluc}}/\partial t$). This is non-zero only when the glucose concentration is increasing, i.e., only when $c_{\mathrm{t}} > 0$. A Hill–type sigmoid response is assumed here too:

$$R_{ins,ph1} = R_{\max,ins1}\frac{\left(\frac{\partial c_{gluc}}{\partial t}\right)^{n_{ins1,gluc}}}{\left(\frac{\partial c_{gluc}}{\partial t}\right)^{n_{ins1,gluc}} + Ct_{Hf,ins1,gluc}^{n_{ins1,gluc}}}\cdot\sigma_{i1,g}\left(c_{gluc}\right) \qquad (A\ 7)$$

with $n_{\mathrm{ins1,gluc}} = 2$, $Ct_{\mathrm{Hf,ins1,gluc}} = 0.03$ mM·s^{-1}, and $R_{\mathrm{max,ins1}} = 21.0\times10^{-5}$ mol·m^{-3}·s^{-1} (Table 1). The Ct_{Hf} value used (0.03 mM/s) is selected so as to give an approximately linear response for a range that likely covers normal physiologic conditions (e.g., 5 mM increase in 10–20 min) as well as dynamic perifusion conditions (e.g., 2–6 mM increases in 1 min). An additional modulating function, $\sigma_{\mathrm{i1,g}}$ is also incorporated in the model to reduce this gradient-dependent response for islets that are already operating at an elevated second-phase secretion rate and to maximize it around c_{gluc} values where islets are likely to be most sensitive ($C_{\mathrm{m}} = 5$ mM) using a derivative of a sigmoid function:

$$\sigma_{i1,g}\left(c_{gluc}\right) = \frac{4c_{gluc}^{4}C_{m}^{4}}{\left(c_{gluc}^{4} + C_{m}^{4}\right)^{2}} \qquad (A\ 8)$$

With all these, total insulin release is obtained as the sum of first- and second-phase releases and an additional modulating function to account for the limiting effect of oxygen availability, which can become important in the core of avascular islets (especially under hypoxic conditions):

$$R_{ins} = \left(R_{ins,ph1} + R_{ins,ph2}\right)\cdot \varphi_{i,o}\left(c_{oxy}\right) \qquad (A\ 9)$$

An abrupt Hill–type modulating function is used as $\varphi_{i,o}(c_{\mathrm{oxy}})$ with $n_{\mathrm{ins,oxy}} = 3$ and $C_{\mathrm{Hf,ins,oxy}} = 3$ μM ($p_{\mathrm{Hf,ins,oxy}} = 2$ mmHg) so that insulin secretion starts becoming limited for local oxygen concentrations that are below ~6 μM (corresponding to a partial pressure of $p_{\mathrm{O_2}} \approx 4$ mmHg). Finally, correct time-scaling of insulin-release could be achieved only with introduction of an extra compartment [1]; without this, insulin responses decreased too quickly compared to experimental observations (~1 min vs. ~5–10 min). Hence, insulin is assumed to be first secreted in a 'local' compartment (Figure 1) in response to the current local glucose concentration (R_{ins}, eq. A9) and then released from there following a first order kinetics, $\mathrm{d}c_{\mathrm{insL}}/\mathrm{d}t = R_{\mathrm{ins}} - k_{\mathrm{insL}}(\mathrm{c}_{\mathrm{insL}} - c_{\mathrm{ins}})$. 'Local' insulin is modeled as an additional concentration with the regular convection model (eq. A1), but having a very low diffusivity ($D_{\mathrm{insL,t}} = 1.0\times10^{-16}$ m^{2}·s^{-1}). The original model, which was calibrated for human islets, used a corresponding rate constant of $k_{\mathrm{insL}} = 0.003$ s^{-1}, corresponding to a half-life $t_{1/2}$ of approximately 4 min [1]. Here, to fit the data obtained with murine islets, this was increased to 0.006 s^{-1} to have a slightly faster release; this was the only parameter modified compared to the original model.

Fluid dynamics

To incorporate media flow, these convection and diffusion models are coupled to a fluid dynamics model. The incompressible Navier–Stokes model for Newtonian flow (constant viscosity) is used for fluid dynamics to calculate the velocity field **u** that results from convection [2, 3]:

$$\rho \frac{\partial \mathbf{u}}{\partial t} - \eta \nabla^2 \mathbf{u} + \rho(\mathbf{u} \cdot \nabla)\mathbf{u} + \nabla p = \mathbf{F}$$
$$\nabla \cdot \mathbf{u} = 0 \qquad \text{(A 10)}$$

Here, ρ denotes density [kg·m^{-3}], η viscosity [kg·m^{-1}·s^{-1} = Pa·s], p pressure [Pa, N·m^{-2}, kg·m^{-1} ·s^{-2}], and **F** volume force [N·m^{-3}, kg·m^{-2}·s^{-2}]. The first equation is the momentum balance; the second one is the equation of continuity for incompressible fluids. Flowing media is assumed to be an essentially aqueous media at body temperature; i.e., the following values are used: T_0 = 310.15 K, ρ = 993 kg·m^{-3}, η = 0.7×10^{-3} Pa·s, c_p = 4200 J·kg^{-1}K^{-1}, k_c = 0.634 J·s^{-1}m^{-1}K^{-1}, α = 2.1×10^{-4} K^{-1}. Incoming media is assumed to be in equilibrium with atmospheric oxygen and, thus, have an oxygen concentration of $c_{oxy,in}$ = 0.200 mol·m^{-3} (mM; corresponding to $p_{O_2} \approx$ 140 mmHg). Inflow velocity is set to v_{in} = 10^{-4} m·s^{-1} (corresponding to a flow rate of 0.1 mL/min in a ~4 mm tube), and along the inlet, a parabolic inflow velocity profile is used: $4v_{in}s(1-s)$, s being the boundary segment length.

Model implementation

The model is implemented in COMSOL Multiphysics 4.4 (COMSOL Inc., Burlington, MA) and solved as a time-dependent (transient) problem, allowing intermediate time-steps for the solver. Computations were done with the Pardiso direct solver as linear system solver with an imposed maximum step of 0.5 s, which was needed to not miss changes in the incoming glucose concentrations that could be otherwise overstepped by the solver. Calculations are done with two spherical islets of 100 and 150 μm diameter placed in a 2D cross-section of a cylindrical tube with fluid flowing from left to right (Figure 3 illustrates the geometry used). Islets were considered homogeneous inside; individual cells (e.g., α- or β-cells) were not considered separately. Islet sizes were selected based on our analysis of the size distribution of isolated islets [17]. This confirmed that the expected value of islet diameter is 95 μm, whereas the expected

value of islet volume is 1.2×10^6 μm^3, corresponding to the volume of an islet with d = 133 μm [17]. As initial conditions, atmospheric oxygen (0.200 mol·m^{-3}), low glucose (3 mM), and zero insulin concentrations were assumed inside the perifusion chamber. Stepwise increments in the incoming glucose concentration were implemented using the smoothed Heaviside step function at predefined time points t_i, $c_{\text{gluc}} = c_{\text{low}} + \Sigma c_{\text{step,i}} \cdot \texttt{flc1hs}(t - t_i, \tau)$. For FEM, COMSOL's predefined 'Extra fine' mesh size was used. In the convection and diffusion models, the following boundary conditions were used: insulation/symmetry, $\mathbf{n}\cdot(-D\nabla c + c\mathbf{u}) = 0$, for walls, continuity for islets. For the outflow, convective flux was used for insulin, glucose, and oxygen, $\mathbf{n}\cdot(-D\nabla c) = 0$. For the inflow, inward flux was used for all components with zero for insulin ($N_0 = 0$), $c_{\text{gluc}} \cdot v_{\text{in}}$ for glucose, and $c_{\text{oxy,in}} \cdot v_{\text{in}}$ for oxygen. In the incompressible Navier-Stokes model, no slip ($\mathbf{u} = 0$) was used along all surfaces corresponding to liquid-solid interfaces. For the outlet, pressure, no viscous stress with $p_0 = 0$ was imposed.

References (for Appendix 1)

1. Buchwald P: **A local glucose-and oxygen concentration-based insulin secretion model for pancreatic islets.** *Theor Biol Med Model* 2011, **8:**20.

2. Truskey GA, Yuan F, Katz DF: *Transport Phenomena in Biological Systems.* Upper Saddle River, NJ: Pearson Prentice Hall; 2004.

3. Comsol, AB: *COMSOL Multiphysics Modeling Guide, version 3.4.* COMSOL AB; 2007.

4. Riley KF, Hobson MP, Bence SJ: *Mathematical Methods for Physics and Engineering. A Comprehensive Guide.* Cambridge: Cambridge University Press; 1997.

5. Hill AV: **The possible effects of the aggregation of the molecules of haemoglobin on its dissociation curves.** *J Physiol* 1910, **40:**iv-vii.

6. Hill AV: **The combinations of haemoglobin with oxygen and with carbon monoxide.** *Biochem J* 1913, **7:**471-480.

7. Goutelle S, Maurin M, Rougier F, Barbaut X, Bourguignon L, Ducher M, Maire P: **The Hill equation: a review of its capabilities in pharmacological modelling.** *Fundam Clin Pharmacol* 2008, **22:**633-648.

8. Michaelis L, Menten ML: **Die Kinetik der Invertinwirkung.** *Biochem Z* 1913, **49:**333-369.

9. Henquin JC, Dufrane D, Nenquin M: **Nutrient control of insulin secretion in isolated normal human islets.** *Diabetes* 2006, **55:**3470-3477.

10. Dionne KE, Colton CK, Yarmush ML: **Effect of hypoxia on insulin secretion by isolated rat and canine islets of Langerhans.** *Diabetes* 1993, **42:**12-21.

11. Buchwald P: **FEM-based oxygen consumption and cell viability models for avascular pancreatic islets.** *Theor Biol Med Model* 2009, **6:**art. 5.

12. Comsol, AB: *COMSOL Multiphysics User's Guide, version 3.4.* COMSOL AB; 2007.

13. Avgoustiniatos ES, Colton CK: **Effect of external oxygen mass transfer resistances on viability of immunoisolated tissue.** *Ann NY Acad Sci* 1997, **831:**145-167.

14. Martin Y, Vermette P: **Bioreactors for tissue mass culture: design, characterization, and recent advances.** *Biomaterials* 2005, **26:**7481-7503.

15. Rorsman P, Eliasson L, Renstrom E, Gromada J, Barg S, Gopel S: **The cell physiology of biphasic insulin secretion.** *News Physiol Sci* 2000, **15:**72-77.

16. Ritzel RA, Veldhuis JD, Butler PC: **Glucose stimulates pulsatile insulin secretion from human pancreatic islets by increasing secretory burst mass: dose-response relationships.** *J Clin Endocrinol Metab* 2003, **88:**742-747.

17. Buchwald P, Wang X, Khan A, Bernal A, Fraker C, Inverardi L, Ricordi C: **Quantitative assessment of islet cell products: estimating the accuracy of the existing protocol and accounting for islet size distribution.** *Cell Transplant* 2009, **18:**1223-1235.
