# Supplementary figures and images for: Experimental evaluation and computational modeling of the effects of encapsulation on the time-profile of glucose-stimulated insulin release of pancreatic islets
Source: Biomed Eng Online. 2015 Mar 28;14:28. doi: 10.1186/s12938-015-0021-9 (PMC4403786; doi:10.1186/s12938-015-0021-9)

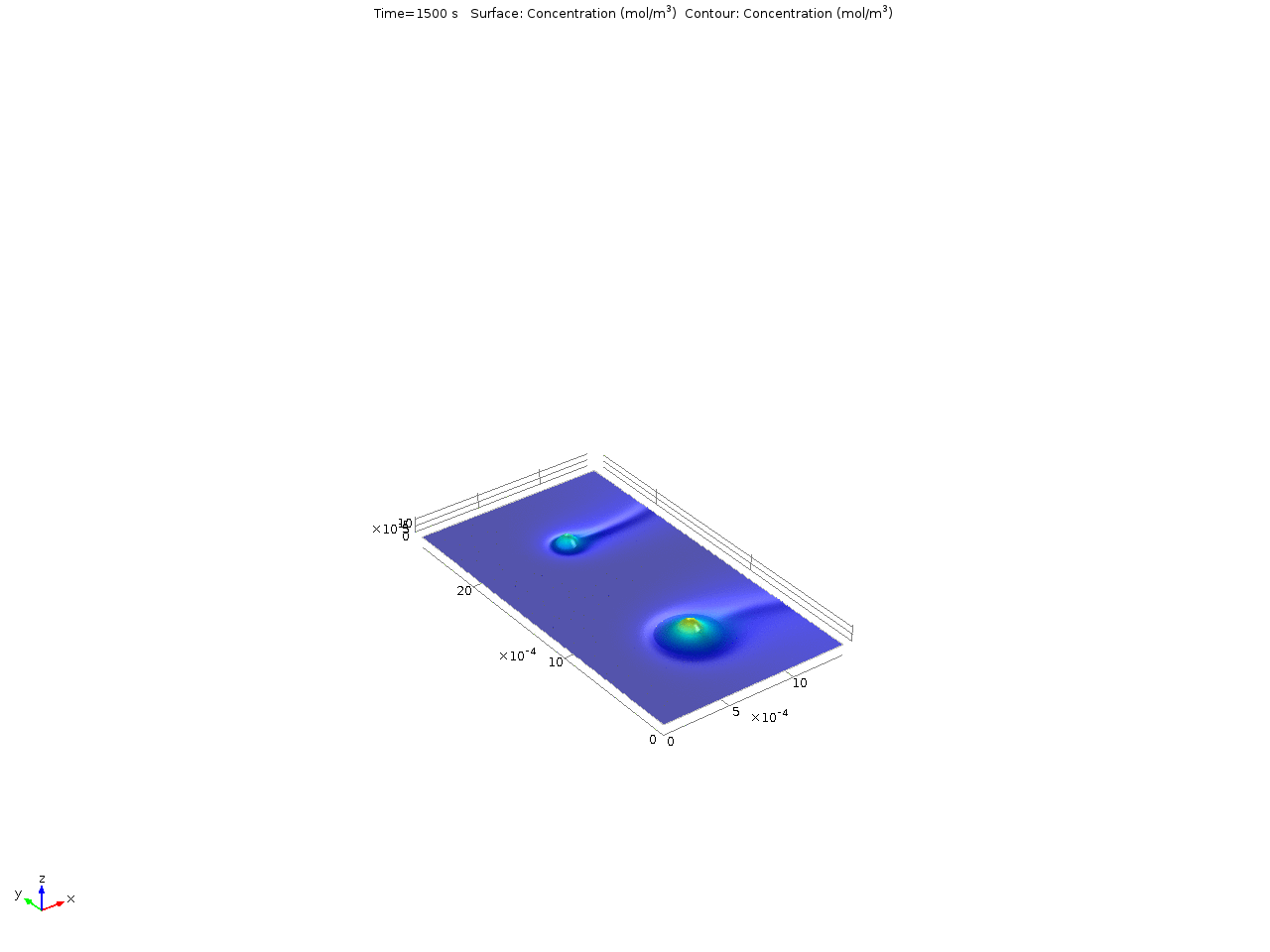

Supplement: Additional file 3: — Animated GIF A2: GlucInsDyn_wHalfEncaps_wParamGeo_Model20_v44_InsConc01.gif. Movie (.avi) and corresponding animated image (.gif) files showing the parallel comparison of the time-course of the GSIR response of free and encapsulated islets (both having d = 150 μm; encapsulated islet on the right side) to a glucose step (3 mM → 11 mM → 3 mM). The same 3D surface representation with insulin concentrations as height data and oxygen concentration as color code (blue = high, red = low) is used as in Figure 7. [file 12938_2015_21_MOESM3_ESM.gif]
